# UV LED Curing for Silicone Hydrogel Contact Lenses: Breakthrough in Curing Properties and Cosmetic Characteristics

**DOI:** 10.3390/polym17212834

**Published:** 2025-10-24

**Authors:** Saravanan Nanda Kumar, Nadia Adrus, Jamarosliza Jamaluddin, Farahin M. Mizi, Fatria Syaimima Saiful Azim, James Jeyadeva Govindasamy

**Affiliations:** 1 Department of Chemical Engineering, Faculty of Chemical and Energy Engineering, Universiti Teknologi Malaysia (UTM), Johor Bahru 81310, Johor, Malaysia; saravanan.nanda_kumar@alcon.com (S.N.K.); jamarosliza@utm.my (J.J.); farahinmizi@gmail.com (F.M.M.); fatriaazim@gmail.com (F.S.S.A.); 2 Alcon (Ciba Vision Johor Sdn Bhd), Gelang Patah 81560, Johor, Malaysia; james_jeyadeva.govindasamy@alcon.com; 3 IJN-UTM Cardiovascular Engineering Centre, Institute of Human Centered Engineering, Universiti Teknologi Malaysia (UTM), Johor Bahru 81310, Johor, Malaysia; 4 Institute of Bioproduct Development, Universiti Teknologi Malaysia (UTM), Johor Bahru 81310, Johor, Malaysia

**Keywords:** UV LED curing, silicone hydrogel contact lens, high-performance lens production, degree of conversion, cosmetic characteristics

## Abstract

Ultraviolet light-emitting diode (UV LED) technology offers advantages over conventional UV mercury (UV Hg) lamps, including precise wavelength control, high energy efficiency and rapid curing. While UV LED is widely applied in sectors like dentistry, printing, and electronics, its application in contact lens manufacturing remains relatively low. This study evaluates the feasibility of integrating UV LED technology curing as a replacement for UV Hg lamps to produce silicone hydrogel contact lenses. Many manufacturers utilizing UV Hg systems encounter challenges such as extended curing times and increased cosmetic defect rates. In this study, lenses were formulated using a mixture of hydrophobic macro-monomer, silicone monomer, and hydrophilic monomer. The formulations were cured using both UV LED and UV Hg lamps systems under controlled intensities, and two curing configurations were assessed: single-sided (SC) and double-sided (DC). The UV Hg light intensity was maintained between 1.1 and 3.1 mW/cm^2^, reflecting standard production values, while the UV LED intensity was set at 32 mW/cm^2^ to ensure uniform light distribution in the mold. The findings showed an improved degree of conversion (DOC) for UV LED cured lenses (86–88%) compared to UV Hg (79.5–82.3%), along with increased water content (ranging between 34 and 36.8%) and ion permeability (7.1–8.3 mm^2^/min). The optical properties of the cured lenses remained consistent across both methods. Notably, UV LED curing reduced cosmetic defects by up to 50% and shortened curing time by 3 to 4 times. These enhancements support UV LED as a superior alternative for contact lens curing, enabling scalable, efficient, and high-quality manufacturing.

## 1. Introduction

Silicone hydrogel lenses have emerged as a major advancement in the field. They often incorporate macromonomers as the structural backbone and hydrophilic polymers within their crosslinking networks to improve oxygen permeability, flexibility, and comfort [1]. These innovations reduce phase separation, improve wettability, and support extended wear, marking a major advancement in contact lens material science. These materials provide superior oxygen permeability and surface wettability compared to traditional hydrogels, significantly improving wearer comfort [2,3,4].

In commercial manufacturing, silicone hydrogel lenses are commonly produced using the mold casting technique, in which a liquid monomer formulation is cured between a convex and a matte concave mold. This photopolymerization process is conventionally initiated by ultraviolet mercury (UV Hg) light, which polymerizes the lens material into its final form [5,6]. While this method has been shown to produce lenses that meet basic industrial standards in terms of refractive index, water content, and lens geometry, it requires long curing times, is associated with inconsistent polymerization, and often results in a higher frequency of cosmetic defects such as surface damage, tears, and irregularities.

As various industries, including coatings, flooring, and dental materials, transition toward ultraviolet light emitting diode (UV LED) technology, the advantages of this approach have become increasingly apparent. UV LEDs offer precise wavelength control, rapid curing, reduced energy consumption and longer operational lifespans [7,8,9]. Emissions in the 365–405 nm range enable efficient, monochromatic polymerization, which has been successfully applied in other photopolymerization based systems [10,11].

Despite these advantages, the use of UV LED curing in contact lens manufacturing remains relatively low. Most previous studies have focused on the effect of UV LEDs on general hydrogel materials and lab scale biomedical polymers rather than industrial scale silicone hydrogel lens production. For instance, Ayub et al. [12] discussed the efficiency of UV LED curing in hydrogel synthesis. Unlike UV mercury lamps, UV LEDs emit concentrated light at 365 nm, enabling effective photopolymerization, which is ideal for contact lens and dental applications. A few studies have systematically evaluated how UV LED curing influences key lens properties such as the degree of conversion (DOC), water content, ion permeability, refractive index, or lens metrology under realistic manufacturing conditions [7]. Moreover, while UV Hg curing has historically been applied in a single sided (SC) configuration, the potential benefits or drawbacks of a double-sided (DC) UV LED curing approach have not been fully explored.

The studies on UV Hg curing generally report that SC exposure can result in uneven energy distribution, especially in thick or optically dense materials, which may lead to incomplete curing in deeper regions of the lens. This can contribute to residual monomers, compromised mechanical integrity, or visual defects [8,9,13]. However, the influence of dual sided UV exposure on curing uniformity, material crosslinking, and surface integrity, particularly in silicone hydrogel systems, remains poorly understood. There is a significant knowledge gap regarding how SC and DC UV LED configurations impact not only polymerization efficiency but also the cosmetic and physical characteristics of the finished lenses. To address these gaps, this study evaluates the feasibility of UV LED curing as a sustainable and high-performance alternative to UV Hg curing in silicone hydrogel contact lens manufacturing. Using a commercially relevant acrylamide-based formulation and existing photoinitiators, lenses were cured using both SC and DC UV LED configurations. The performance indicators were systematically assessed and benchmarked against lenses cured with conventional UV Hg systems. In particular, the study focused on reducing curing time, enhancing physical and optical properties, and minimizing cosmetic defects, all while ensuring compliance with industrial specifications.

Ultimately, this research bridges a critical gap between laboratory scale UV LED hydrogel studies and their application in full scale contact lens production. The findings support the integration of green, efficient curing technologies in ophthalmic device manufacturing and present a pathway toward environmentally responsible lens production

## 2. Materials and Methods

### 2.1. Curing of Contact Lenses Using UV Hg and UV LED

The Lotrafilcon B formulation used in this study was produced by the contact lens manufacturing company, Ciba Vision Johor Sdn Bhd, Gelang Patah, Johor, Malaysia, where the research was conducted. The formulation comprises a hydrophobic macromonomer, silicone monomer, hydrophilic monomer, photoinitiator, and an alcohol-based solvent. The curing of contact lenses involves the transformation of the liquid monomer formulation into a polymer with a semi-solid consistency. The formulation was cured using two light sources: (1) a UV Hg oven from the existing manufacturing lines, and (2) a UV LED light source model LEDLINE 500 manufactured by Dr. Hoenle AG, Germany, which was installed into a prototype oven. The curing parameters are shown in Table 1.

Additionally, two curing configurations were tested: single sided (SC) and double sided (DC) curing. In the SC configuration, the liquid lens monomer mixture, sealed within its mold, was exposed to UV LED light emitting from the top of the curing oven. The DC configuration involved exposing the capped monomer mixture in its mold to UV LED sources positioned at both the top and the bottom of the curing oven. The mold was produced internally in the mold production section of the company’s manufacturing facility. This process was validated and fully automated, ensuring consistent mold dimensions. The dispensing volume of the formulation into the mold was an automated process controlled by a PLC system. During curing, the molds were sealed, which restricted airflow.

**Figure 1:**
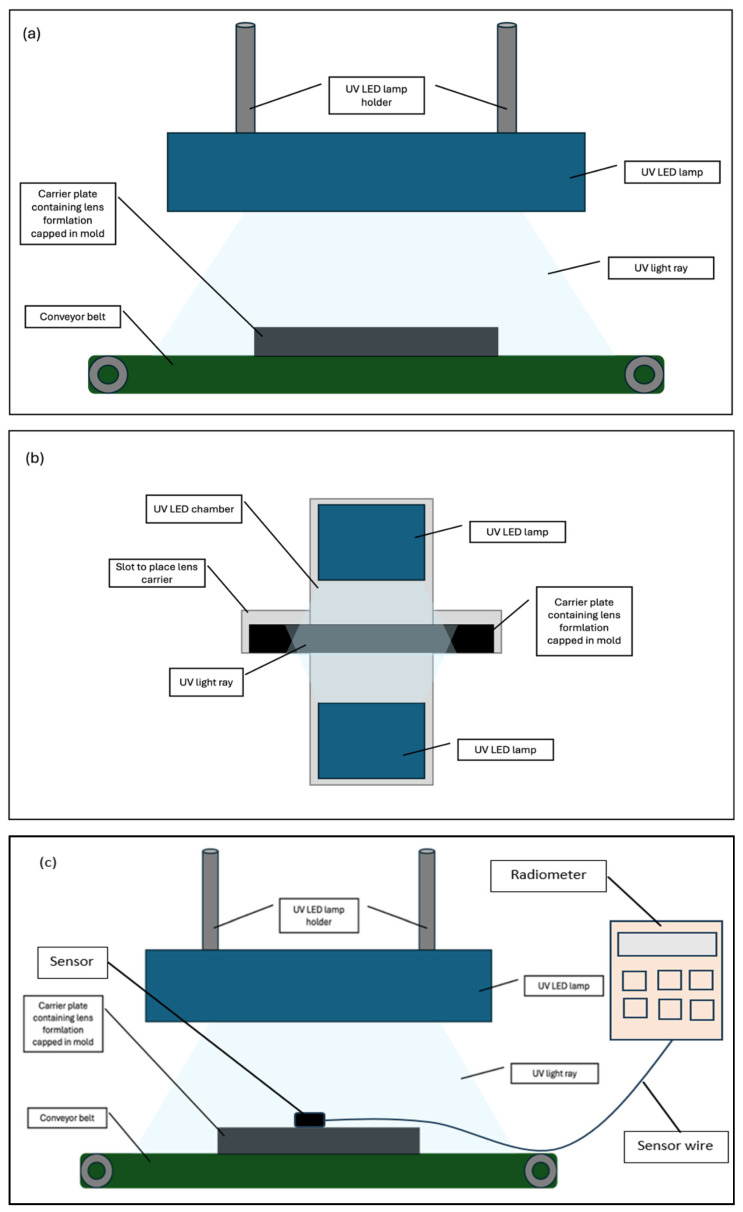
UV LED curing configurations. (**a**) UV LED SC for lens samples on moving conveyor belt. (**b**) UV LED DC for lens samples in static curing chamber. (**c**) UV LED light intensity measurement using Radiometer at static condition prior to commencing curing process.

UV mercury light intensity was monitored during production using a PLC-based system. A sensor located beneath the light source in the curing chamber captured the intensity, which was shown on the machine’s control panel and automatically recorded by the PLC software RSLogixs500 version 11. The intensity used in this study followed standard process parameters, ranging from 1.1 to 3.1 mW/cm^2^ across the UVA spectrum. This measurement method was integrated into the process. For UV LED light, intensity was measured using a Radiometer Model 7859C from Dr. Hoenle AG, Germany. The sensor probe was placed on top of the carrier plate—holding the formulation inside the mold—before curing began. The reading appeared on the Radiometer’s control panel. Figure 1 shows the schematic diagram of the SC and DC curing setups and the UV LED intensity measurement method.

The distance between the UV mercury lamp and the carrier plate containing the lens formulation capped in the mold was maintained at 7 cm, resulting in an intensity range of 1.1 to 3.1 mW/cm^2^. To ensure consistency with current manufacturing conditions, the same distance was applied in the UV LED study conducted using the prototype oven, resulting in an intensity of 32 mW/cm^2^.

This research addresses a real industrial challenge in UV photopolymerization curing for contact lenses, where a fixed UV Mercury lamp setup (1.1–3.1 mW/cm^2^ at 7 cm distance) leads to quality issues if intensity deviates—under-curing below 1.1 mW/cm^2^ causes lens adhesion and tearing, while over-curing above 3.1 mW/cm^2^ results in heat-induced defects due to insufficient cooling. To improve curing efficiency, the study explores UV LED technology using a lab-scale prototype that mimics production conditions, which intends to identify optimal curing at 32 mW/cm^2^ without compromising lens quality, offering a practical path for industrial adoption.

### 2.2. Characterization of UV Hg and UV LED Post-Cured Finished Lenses

#### 2.2.1. Degree of Conversion Based on Functional Group

The functional groups of liquid formulation and post-cured contact lenses were characterized using a Fourier Transform Infrared Spectrophotometer (FTIR) (Thermo Fisher Scientific’s Nicolet 170 SX, manufactured by Thermo Fisher Scientific Inc., Waltham, MA, USA) with the attenuated total reflection (ATR) technique. The type of crystal used was diamond. This type of crystal is known to have sensitive detectability of functional groups in the Lotrafilcon B formulation. The wavenumber spectra ranged between 2000 and 800 cm^−1^ with a total of 64 scans per analysis. The degree of conversion (DOC) was determined by comparing the absorption bandwidths between the contact lens liquid formulation and the post-cured contact lenses. Based on the spectral data, the area under the peak within 1635 cm^−1^ to 1728 cm^−1^ corresponded to the C=C and C=O functional groups of the lens formulation mixture. The DOC of the final cured contact lenses was determined using Equation (1) [14,15], based on the testing of 3 lens samples per curing time to obtain the average value.

(1) $$\%DOC=100-\left[\frac{(Area\;below\;peak\;at\;1635{\;cm}^{-1}/Area\;below\;peak\;at\;1728{\;cm}^{-1})\;post-cured\;lens}{(Area\;below\;peak\;at\;1635{\;cm}^{-1}/Area\;below\;peak\;at\;1728{\;cm}^{-1})\;liquid\;formulation}\times 100\right]$$

#### 2.2.2. Gel Fraction

Gel fraction was determined using the percentage of non-volatile extractables method. A known amount of the polymer sample was weighed before extraction, recording the initial weight (W_i_). The polymer sample was then subjected to a 2-propanol solvent extraction process to remove the non-volatile extractables, ensuring that all soluble components were removed without degrading the polymer. The extraction was performed for 48 h. After the extraction, the sample was dried to remove any residual solvent, typically using a vacuum oven or another suitable drying method. The dried sample was then weighed to obtain the final weight (W_f_). The gel fraction was calculated using Equation (2) [16].

(2) $$Gel\;Fraction\left(\%\right)=(\frac{W_{f}}{W_{i}})\times 100\%$$

#### 2.2.3. Refractive Index and Water Content

The refractive index (RI) was determined using a benchtop refractometer (Reichert Mark III model, manufactured by Reichert Technologies Inc., Depew, NY, USA). Since the measurements were temperature-dependent, all tests were performed after equilibrating the lenses at 25 ± 0.2 °C for 2 h in saline solution. To perform the measurement, the upper prism cover was lifted and approximately 2 mL of saline was placed on the sample stage. A lens was then carefully placed, with the base curve facing up, onto the center of the sample stage. An additional 2 mL of saline was added around or on top of the lens to ensure sufficient surface contact. Once the system stabilized at the correct temperature, the RI was recorded, and the device automatically calculated and displayed the water content percentage. The percentage of water content was auto calculated and displayed along with the RI reading. A total of 30 lens samples were tested, and the average results were analyzed.

#### 2.2.4. Ion Permeability

Ionic permeability of the samples was evaluated using a custom-designed two-chamber diffusion cell. The system consisted of a donor chamber filled with 16 ± 0.5 mL of 0.1 N NaCl solution and an acceptor chamber containing 32 mL of ultra-purified water (UPW). Hydrated disks (12 mm in diameter), both before and after sterilization, were positioned between the two chambers over a 10 mm diameter orifice to ensure a defined diffusion area. The conductivity of the solution in the acceptor chamber was continuously monitored using a Consort D230 conductivity meter at a controlled temperature of 32 °C. For each condition, three independent measurements were conducted.

The measured conductivity values were converted into NaCl concentrations using a previously established calibration curve. The ionic permeability (D ion) was then calculated using the following equation:

(3) $$\frac{F\times V}{A}=D_{ion}\frac{dc}{dx}$$

where F is the rate of ion transport (mol/s), V is the volume of the acceptor chamber (cm^3^), A is the cross-sectional area of the sample exposed to diffusion (cm^2^), and $\frac{dc}{dx}$ is the initial concentration gradient of NaCl across the membrane (mol/cm^3^/cm).

#### 2.2.5. Lens Metrology

The lens thickness was measured using the Rehder Thickness Gauge Model ET-3, manufactured by Createch Rehder Development Company, Greenville, SC, USA. Prior to the measurement, the lens samples were hydrated in UPW for a minimum of 2 min to ensure consistent hydration. A minimum hydration time of 2 min was required for thickness parameter measurement to ensure consistent hydration across all test samples. Minimum hydration was sufficient, as the lens tested were fully equilibrated prior to the measurement. After soaking, excess surface moisture was gently removed by placing each lens on a blotting cloth. Thickness measurements were then performed at five different points across the lens: the center, top, bottom, left, and right edges, to account for uniformity across the lens circumference. In addition to thickness, lens diameter, center thickness and base curve equivalent (BCE) were measured using J&L Replacement metrology equipment model EVHB-M3. All measurements were conducted in a controlled environment with the temperature maintained within 22.5–23.5˚C to ensure accuracy and reproducibility.

#### 2.2.6. Cosmetic Quality Check

Lens cosmetic quality inspection was performed through a visual inspection method with custom-made high magnification equipment. The lens cosmetic quality inspection was a 100% in-process screening. The most common defects inspected during cosmetic inspection included holes, inclusions, flash, flash particles, irregular edges, torn-in-molds, surface damage, tears, and the presence of wrinkled spots.

## 3. Results

### 3.1. Significance of Improved Degree of Conversion and Gel Fraction

**Figure 2:**
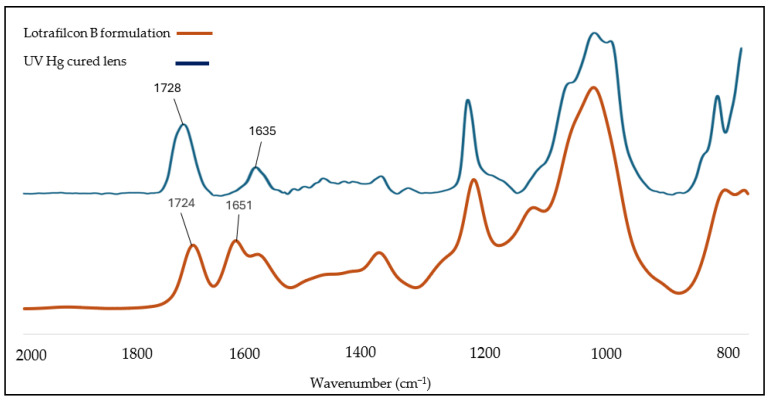
FTIR spectra for both Lotrafilcon B before curing and UV Hg cured lens samples after 30 min curing.

Figure 2 presents the FTIR spectra of Lotrafilcon B, along with a representative lens spectra of a lens cured using UV Mercury (Hg) lamps, as per the intensity and duration parameters outlined in Table 1. The spectral region between 1635 cm^−1^ and 1728 cm^−1^ corresponds to the characteristic absorption bands of the C=C and C=O functional groups present in the lens formulation mixture.

**Figure 3:**
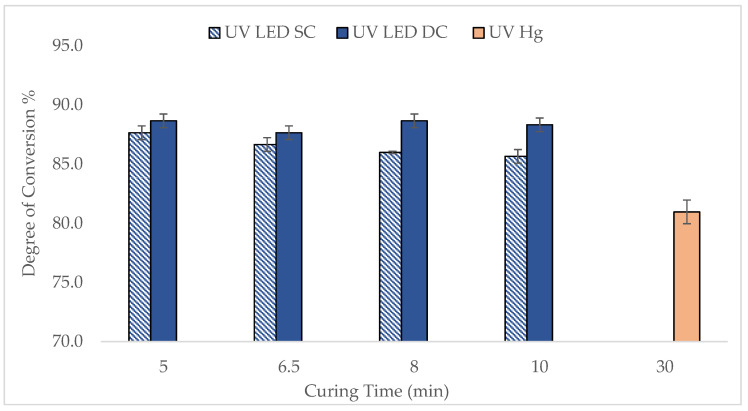
Comparison of DOC of lenses cured between UV LED and UV Hg at various curing times for both SC and DC setups.

Figure 3 illustrates the DOC of UV LED-cured lenses in both SC and DC configurations across various curing time. The findings were also compared with the lenses produced by UV Hg lamp. DOC was measured directly after curing, capturing both cured and uncured components.

In general, the DOC of lenses cured by UV LED, particularly in the DC configuration, was higher than that achieved with UV Hg, demonstrating better efficiency of the UV LED curing. Specifically, the DOC for the UV LED DC lenses ranged between 88 and 89%, slightly higher than the 86% to 88% observed for UV LED SC lenses, indicating that double-sided curing marginally improves polymerization. Notably, the DOC for UV LED lenses reached a high level within just 5 min, whereas the UV Hg lens only reached 79.5–82.3% DOC after 30 min. The trend does not reduce gradually but there is no significant difference in DOC as a function of UV time. The rapid curing observed with UV LEDs is consistent with findings reported in the literature by Ayub et al. [12] whereby a custom water-soluble photoinitiator (WSPI) was developed to match the LED’s wavelength, achieving over 90% monomer conversion, as high photon energy at a monochromatic wavelength of 365 nm contributed to faster and more efficient photopolymerization. Beyond 5 min, the DOC remained relatively stable, which is consistent with the limited mobility of polymer chains at higher viscosities, which slow reaction rates over time. To further validate these findings, the lenses were subjected to gel fraction (GF) analysis after the extraction process. An extraction step was carried out as a part of the downstream lens manufacturing process using isopropyl alcohol (IPA) and ultra-pure water (UPW). This step removes unreacted monomers, short-chain oligomers, and uncrosslinked polymers, resulting in a higher gel fraction value during testing

It is important to note that the GF data showed values at around 98–99%, indicating that a highly crosslinked, insoluble network was effectively formed for curing durations exceeding 10 min. This suggests that, despite the early high DOC, achieving full crosslinking and optimal physical stability may require longer curing times of around 10 min to ensure complete network development. Compared to UV mercury curing, lenses cured using UV LED showed a higher gel fraction value, typically between 96 and 97%, indicating that UV LED curing offers a more effective option.

Overall, both DOC and GF data demonstrate that the lens formulation was successfully polymerized and crosslinked using UV LED technology [6], and these parameters serve as reliable indicators of the effectiveness of the polymerization process. However, it should be noted that other important properties also play a crucial role in determining the optimal curing parameters, which will be discussed further in the next subchapter.

### 3.2. Physical Properties of Lenses

**Figure 4:**
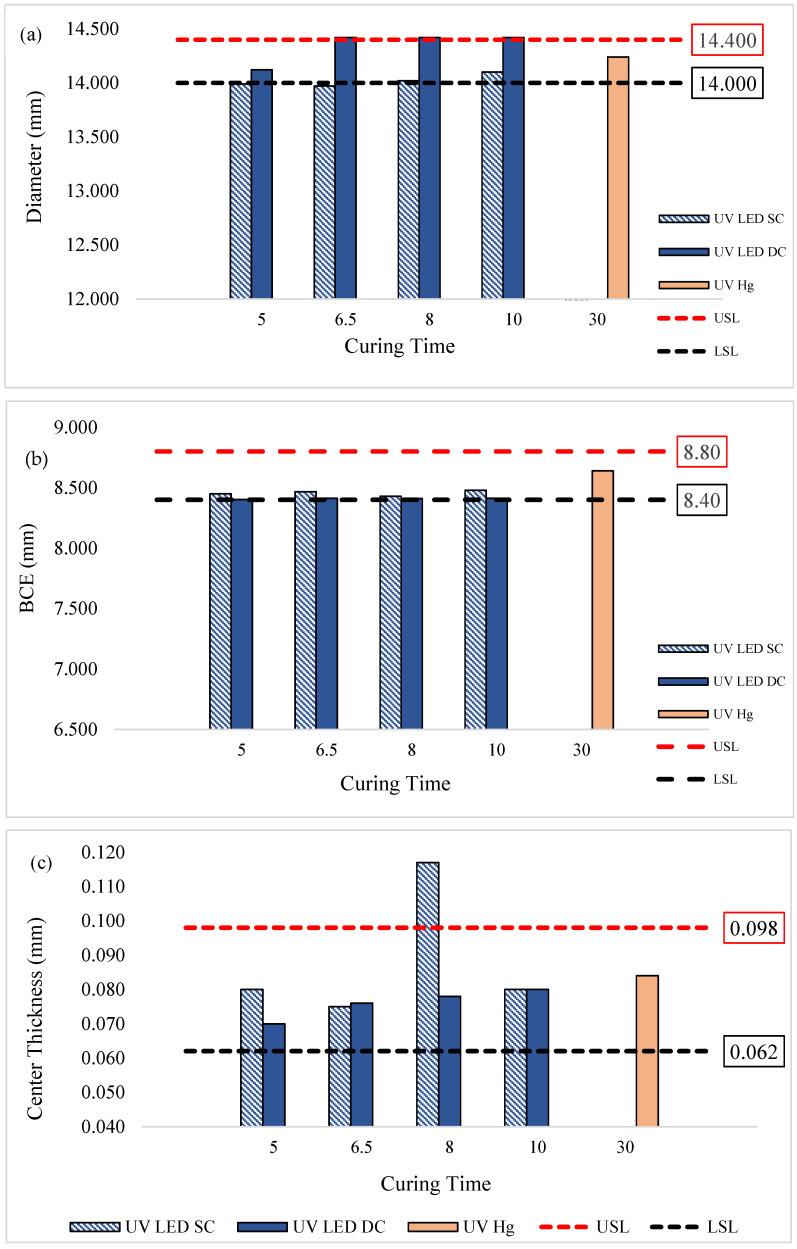

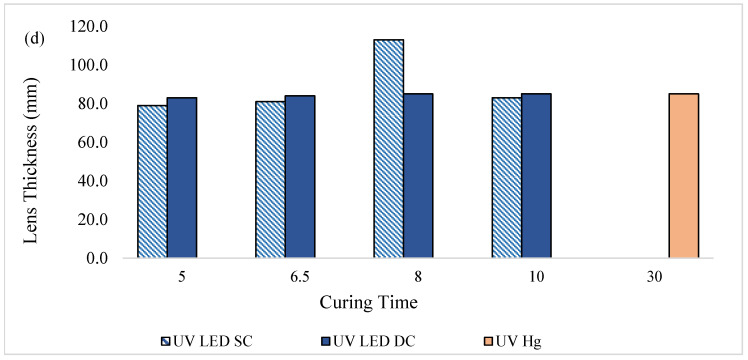
Physical properties of lenses cured by UV LED SC, UV LED DC, and UV Hg at various curing times. (**a**) Diameter of lenses; (**b**) base curve equivalent (BCE); (**c**) center thickness; (**d**) lens thickness.

The physical properties of the lenses, including measurements of lens diameter, base curve equivalent (BCE), lens thickness, and center thickness, were assessed to evaluate the accuracy of lens metrology and their dimensional consistency. Figure 4a shows the diameter profile of lenses cured by UV LED compared to UV Hg. Generally, the lenses cured by UV LED in the SC configuration tend to approach the lower specification limits (LSL), with diameters only meeting the LSL after curing for 8 min or more. This suggests that curing times of less than 8 min may be insufficient for the formulation to achieve uniform curing. Consequently, the lens may be unable to maintain its shape and could experience slight shrinkage upon removal from the mold [17].

In contrast, UV LED in the DC configuration yielded lenses with diameters mostly above the upper specification limits (USL). This could be attributed to the targeted UV emission wavelength of the LED lamp, which delivers intense energy from top and bottom curing, resulting in rapid polymerization. Such rapid curing may trap unreacted monomers within the crosslinked networks, resulting in lenses that are less firm and more prone to flattening and enlargement when removed from the mold [18]. These findings are supported by the BCE data in Figure 4b, where BCE values were prone to be near the LSL, indicating a tendency toward flatter lenses compared to those cured by UV Hg.

These physical property observations align with previous research, such as Young et al. [19], which emphasizes that changes in lens diameter necessitate corresponding adjustments in BCE to maintain a proper fit. Despite the reductions in diameter and BCE observed here, these variations are not concerning; smaller BCE values and diameters can facilitate better tear exchange and oxygen flow. Additionally, studies have indicated that a 0.4 mm change in BCE generally corresponds to a 0.2 mm change in diameter, which needs to be limited in order to sustain similar on-eye fit [20,21]. Therefore, the approximately 0.2 mm reduction in BCE in our UV LED-cured lenses aligns with an approximate 0.1 mm decrease in diameter, which remains within acceptable fitting ranges. Figure 4c and Figure 4d show the lens center thickness and overall lens thickness, respectively.

Generally, all lenses cured by UV LED in both the SC and DC configurations met the specified center thickness, and their overall thickness were comparable to those cured by UV-Hg. However, the lenses cured by UV LED SC at 8 min exhibited a notable deviation. This variation was likely due to the mold used for curing, which was designed for lenses with higher optical addition (+) power, contributing to elevated thickness and center thickness values. Since lens thickness and center thickness are influenced by the optical power properties, which were not strictly controlled in this study, some variations in these parameters were expected.

### 3.3. Refractive Index and Water Content of UV-Cured Contact Lens

**Figure 5:**
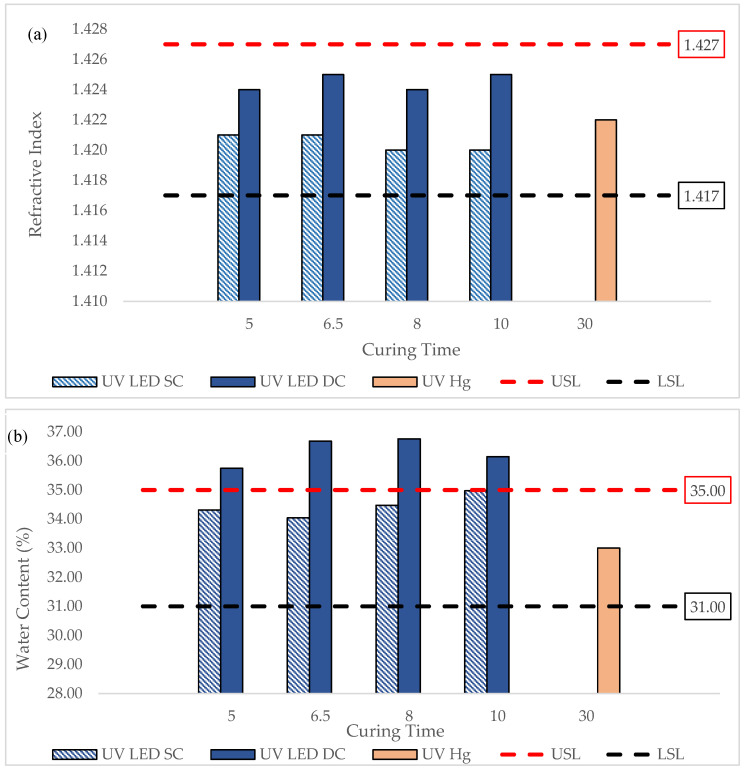
(**a**) Refractive index (RI), and (**b**) water content (WC) of lenses cured by UV LED and UV Hg at various curing times.

Figure 4 presents the refractive index (RI) with the corresponding water content of lenses cured by UV LED and UV Hg. As shown in Figure 5a, the RI of lenses cured under both SC and DC configurations fell within the specification range and were comparable to UV Hg-cured lenses. Notably, the DC-cured lenses exhibited a slightly higher RI than those cured with UV LED in SC configuration or with conventional UV Hg lamps. In line with the higher DOC observed in DC-cured lenses in Section 3.1, this elevated RI may be associated with increased crosslinking density and more complete polymerization at the surface. According to literature, higher RI also indicates better optical quality of the lenses, which can enhance lens durability and visual clarity [22,23]. Typically, the RI of contact lenses exceeds 1.422, and changes in RI also reflect variations in the equilibrium water content of silicone hydrogel lenses.

Figure 5b shows the water content of all UV-LED-cured lenses, where DC-cured lenses demonstrated higher water content (WC), occasionally exceeding specification limits. This suggests that while the surface regions of DC cured lenses are more tightly polymerized, the internal bulk may remain relatively loosely crosslinked due to rapid outer-layer curing. This finding is similar to those reported in the literature, whereby, in dental composite curing, the outer surface receives the most light, leading to a higher polymerization and crosslinking density, and, as such, the inner bulk remains under-cured or less crosslinked [24,25]. As a result, the lens core can retain more water, especially in lenses with larger diameters and volumes. This dual effect highlights the complex relationship between curing configuration, network structure, and resulting lens properties. This finding is similar to those reported by Jose et al., whereby loosely crosslinked regions in hydrogels can retain more water. In fact, the dual effect of surface rigidity versus internal flexibility is a key consideration in biomedical hydrogel, contact lenses, and drug delivery systems [26]. The water content of the UV-LED-SC-cured lens was within the specification, while the UV-LED-DC-cured lens was prone to exceeding the specification limit. This data can be attributed to the larger lens diameter obtained as shown in Figure 3. A larger diameter results in a higher volume of lens, which then allows more water to be stored within the lens, hence the higher water content.

### 3.4. Ion Permeability of UV-Cured Contact Lenses

Ion permeability (IP) is a critical property in contact lenses, as it directly influences tear exchange, lens movement, and the overall physiological response of the ocular surface [11]. Adequate IP facilitates movement within the ocular environment, supporting the removal of metabolic waste and improved oxygen and ion flow beneath the lens during blinking [27].

**Figure 6:**
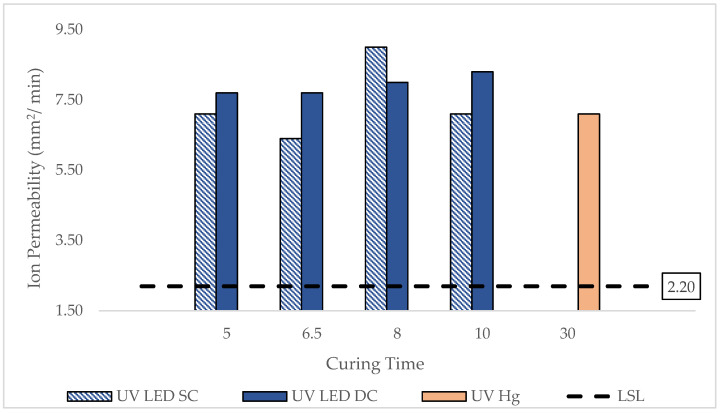
Ion permeability data of lenses cured by UV LED and UV Hg at various curing times.

Figure 6 presents the IP values of silicone hydrogel lenses cured under various conditions. All UV-LED-cured lenses in both SC and DC configurations demonstrated IP values well above the LSL, indicating their suitability for supporting ion transport and tear exchange. The highest IP was recorded under UV LED SC at 9.0 mm^2^/min. The data in Figure 6 suggest that UV LED curing, especially with SC exposure, promotes the formation of a lens matrix with favorable IP characteristics, potentially due to a slightly looser or more hydrated polymer network compared to conventional UV Hg curing.

Previous studies support these findings. Nicolson et al. [28] indicated that the optimal IP should exceed 6.4 × 10^−6^ mm^2^/min when measured using the Ionoflux method or 0.0012 mm^2^/min when measured with the Ionoton method. Peng et al. [29] reported an IP of for various silicone hydrogel lenses, including the discontinued O_2_ Optix, while Gavara and Compañ [30] measured 0.00204 mm^2^/min for Air Optix lenses. Peng et al. [29] reported an IP of approximately 0.001 mm^2^/min for various silicone hydrogel contact lenses, including the discontinued O_2_ Optix lens, while Gavara and Compañ [30] obtained an IP of 0.00204 mm^2^/min for Air Optix lenses using the Ionoton method.

These results reinforce the potential of UV LED curing not only to reduce curing time and improve lens esthetics but also to enhance functional performance by enabling higher ion transport capacity. The findings also highlight that curing configuration and exposure duration have measurable effects on polymer structure, influencing permeability. Despite slightly lower DOC in some cases, single-sided curing may produce a network structure more favorable for ion mobility. This underscores the importance of balancing polymerization efficiency with material functionality when optimizing UV LED curing protocols.

### 3.5. Cosmetic and Structural Defects Analysis

Ensuring high product quality through defect analysis is a fundamental aspect of contact lens manufacturing. This process aims to identify and reduce defects, supporting good manufacturing practices that require 100% inspection to prevent substandard lenses from reaching consumers. Only lenses that pass strict quality control are packaged, safeguarding the manufacturer’s reputation and consumer safety.

**Figure 7:**
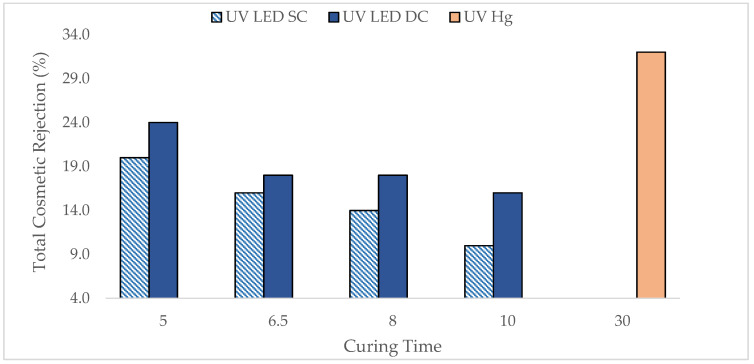
Total curing-related cosmetic rejections of lenses cured by UV LED and UV Hg at various curing times.

Figure 7 depicts the curing related cosmetic rejections from the lenses produced by UV LED and UV Hg. Generally, the rejection of lenses cured by UV LED decreased as curing time increased from 5 to 10 min. Notably, the UV-LED-cured lenses showed a 20–50% improvement compared to UV Hg, starting at around 8 min for UV LED SC and 6.5 min for UV LED DC. This trend strongly supports the observation seen in DOC as demonstrated in Figure 1. Prolonged curing time is favorable to allow the formulation to achieve optimal curing, provide adequate crosslinking between polymer chains, enhance the strength of the lenses, and, thus, minimize the strength-related defects during the manufacturing process [31,32].

Table 2 provides a detailed breakdown of cosmetic defects associated with the curing process, including torn-in-mold, surface damage, tears, and wrinkled spots. Variations in the number of defects were observed when comparing lenses cured by UV LED SC and DC. Specifically, the UV LED SC configuration produced lenses with slightly lower defects, especially on the ‘surface damage’ type of defect. This is likely due to more uniform and controlled polymerization, though a slightly longer curing time was needed. On the other hand, the DC configuration, with its more intense energy input from double-sided UV exposure, resulted in more rapid and incomplete polymerization, trapping unreacted monomers within the polymer networks and leading to higher defects. Importantly, the reduction in defects, particularly for ‘tears’ and ‘torn-in-mold’  was up to 50% in UV LED-cured lenses compared to UV Hg, demonstrating the high efficiency of UV LED technology in improving the strength of lenses [33,34].

In summary, the defect analysis confirms that optimized UV LED curing, particularly with appropriate exposure times and configurations, can significantly minimize surface and structural defects in contact lenses. These improvements support higher quality of manufacturing and enhanced lens strength, demonstrating the potential of UV LED technology for producing high-quality lenses.

### 3.6. Summary of Key Results and Findings

This study systematically compared the curing effects of UV LED in two configurations (SC and DC) against the conventional UV Hg lamp on silicone hydrogel contact lenses. The results, summarized in Table 3, indicate that optimal curing times are approximately 10 min for UV LED SC and 6.5 min for UV LED DC at an irradiance of 32 mW/cm^2^. Notably, this represents a significant reduction in curing time compared to the 30 min required for UV Hg, achieving an optimal balance between curing efficiency and defect minimization.

Degree of Conversion (DOC) values exceeded 86% for UV LED cured lenses, significantly higher than UV Hg (79.5–82.3%), reflecting improved polymerization. The refractive index (RI) and water content of UV LED lenses were within acceptable ranges, with UV LED DC lenses showing slightly higher RI, likely due to increased crosslinking and hydration.

Ion permeability measurements showed UV LED lenses achieved values of up to 9.0 mm^2^/min, surpassing the baseline specifications and indicating enhanced oxygen and ion flow. The cosmetic defect analysis revealed that UV LED curing, especially in the SC configuration, reduced surface and internal defects by up to 50% compared to UV Hg, with defect types including torn-in-mold, surface damage, tears, and wrinkled spots. Notably, lens defects decreased markedly with longer curing times, supporting the correlation between sufficient polymerization and improved physical properties.

Overall, the data supports UV LED as an effective, environmentally friendly alternative for contact lens curing, capable of producing high-quality lenses with superior physical, optical, and cosmetic characteristics.

## 4. Conclusions

This study demonstrates that UV LED technology holds significant promise as a feasible alternative curing solution to traditional UV Hg lamps for the curing of silicone hydrogel contact lenses. Through systematic evaluation of both SC and DC configurations under realistic industrial conditions, the findings confirm that UV LED not only drastically reduces curing times up to fourfold but also enhances critical lens properties. These include improved polymerization efficiency, optical quality, physical stability, and cosmetic appearance, all while supporting environmentally responsible manufacturing practices. The reduction in curing time and defects leads to higher manufacturing yields and lower production costs, making this technology highly suitable for large-scale industrial application. The ability to consistently produce high-quality lenses with minimal defects underscores the scalability and practical viability of UV LED curing as a revolutionary advancement in biomedical polymer processing.

Determining optimal curing conditions requires balancing both chemical metrics like gel fraction and conversion rate with the final lens properties that meet commercial standards. This study prioritizes practical implementation, showing that a 10 min curing time using UV LED not only maintains lens quality but also significantly reduces cosmetic defects.

Overall, this research not only bridges critical knowledge gaps but also establishes a new standard for sustainable, efficient, and high-quality contact lens production marking a breakthrough that paves the way for future innovations in environmentally responsible ophthalmic manufacturing.

## Figures and Tables

**Table 1:** Curing parameters for UV Hg and UV LED.

Parameters	UV Mercury	UV LED	
UV lamp setup	Single sided as per industry curing process	Single-sided (SC)	Double-sided (DC)
Curing time (mins)	30	5, 6.5, 8 and 10	5, 6.5, 8 and 10
UV intensity (mW/cm^2^)	1.1–3.1	32	32

**Table 2:** Summary of detailed cosmetic defects influenced by curing using UV LED and UV Mercury from 50 lenses inspected.

UV Source	UV LED								UV Mercury
Intensity (mW/cm^2^)	32								1.1–3.0
Curing layout	Single Sided				Double sided				Single Sided
Curing time	5	6.5	8	10	5	6.5	8	10	30
Torn in mold	4	1	1	1	5	2	3	2	5
Surface damage	2	2	2	1	2	2	2	2	4
Tear	2	4	4	3	4	4	4	4	6
Wrinkled Spot	2	1	0	0	1	1	0	0	1

**Table 3:** Comparison of results for UV Mercury and UV LED.

		UV LED SC				UV LED DC				UV Hg
		Curing Time (min)								
Properties	Specs.	5	6.5	8	10	5	6.5	8	10	30
Refractive Index	1.417–1.427	/	/	/	/	/	/	/	/	/
Water Content (%)	31–35	/	/	/	/	/	/	/	/	/
Diameter (mm)	14.00–14.40	X	X	/	/	/	/	X	X	/
Base curve equivalent (mm)	8.40–8.80	/	/	/	/	/	/	/	/	/
Center thickness (mm)	0.062–0.098	/	/	X	/	/	/	/	/	/
Ion Permeability (mm^2^/min)	≥2.2	/	/	/	/	/	/	/	/	/
Cosmetic rejections (%)	-	20	16	14	10	24	18	18	16	32

Note: (/) Met the specification; (X) Out of specification.

## Data Availability

The original contributions presented in this study are included in the article. Further inquiries can be directed to the corresponding author.

## Abbreviations

The following abbreviations are used in this manuscript:

UV LED	Ultraviolet light emitting diode
UV Hg	Ultraviolet light mercury
SC	Single-sided
DC	Double-sided
DOC	Degree of conversion
FTIR	Fourier Transform Infrared Spectrophotometer
ATR	Attenuated Total Reflection technique
GF	Gel Fraction
RI	Refractive Index
IP	Ion Permeability
WC	Water Content
BCE	Base curve equivalent
WSPI	Water-soluble photoinitiator
LSL	Lower Specification Limit
USL	Upper Specification Limit
UPW	Ultra Purified Water
